# Cyanobiont genetic diversity and host specificity of cyanobiont-bearing dinoflagellate *Ornithocercus* in temperate coastal waters

**DOI:** 10.1038/s41598-021-89072-z

**Published:** 2021-05-04

**Authors:** Miran Kim, Dong Han Choi, Myung Gil Park

**Affiliations:** 1 Research Institute for Basic Sciences, Chonnam National University, Gwangju, 61186 Republic of Korea; 2 Honam National Institute of Biological Resources, 99 Gohadoangil, Mokpo, 587262 Republic of Korea; 3 Marine Ecosystem Research Center, Korea Institute of Ocean Science and Technology, 385 Haeyangro, Yeongdogu, Busan, 49111 Republic of Korea; 4 Department of Oceanography, Chonnam National University, Gwangju, 61186 Republic of Korea

**Keywords:** Environmental microbiology, Ocean sciences, Marine biology

## Abstract

Cyanobacteria are ubiquitous in marine environments and play an important role as primary producers. Some cyanobacteria, the so-called cyanobionts (cyanobacterial symbionts), have a symbiotic relationship with unicellular organisms. Among these relationships, in particular, the nature (e.g., genetic diversity, host or cyanobiont specificity, and cyanobiont seasonality) of the cyanobiont-dinoflagellate host consortia remains poorly understood. In this study, 16S rDNA of cyanobionts in 138 single host cells isolated over four seasons from temperate waters were sequenced using the MiSeq platform. Genetic analysis of cyanobionts from the dinoflagellate host *Ornithocercus* revealed that three genetic types of Synechococcales cyanobionts occurred in a wide range of water temperatures (11–24 °C), and their distribution seemed to be closely associated with variations in salinity. Furthermore, a certain degree of host (or cyanobiont) specificity in cyanobionts (or the host) among *Ornithocercus* species as well as among other dinophysoid species (i.e. *Amphisolenia, Citharistes,* and *Histioneis*), was observed. In addition to the Synechococcales cyanobionts, this study identified OTU sequences affiliated with Vampirovibrionales and Chroococcidiopsidales in some *Ornithocercus* cells, suggesting that *Ornithocercus* species are an additional habitat for these bacterial groups.

## Introduction

Symbiosis can be defined as the relationship between two different organisms living together. It is widespread across all taxa and is found in diverse habitats throughout the marine environment. In terms of symbioses at the unicellular level, the relationships between cyanobacterial symbionts (cyanobionts) and protistan hosts are particularly noteworthy, as some nitrogen-fixing cyanobacteria (diazotrophs) play an important role in primary production, especially in nitrogen-limited oligotrophic oceans^1–3^. Cyanobacteria, mostly pico-sized *Synechococcus* and *Prochlorococcus*, are ubiquitously distributed and are the most abundant photosynthetic organisms on Earth, accounting for a quarter of all carbon fixed in marine ecosystems^4–6^. In contrast to free-living marine cyanobacteria, some cyanobionts are known to be responsible for nitrogen fixation rather than carbon fixation in the host^7,8^. However, the physiological functions of most cyanobionts remain unknown. Cyanobionts have been found in numerous protist groups, including dinoflagellates, tintinnids, radiolarians, amoebae, diatoms, and haptophytes^9,10^. Among these cyanobionts, little is known regarding the nature (e.g., genetic diversity, host or cyanobiont specificity, and cyanobiont seasonality) of the symbiosis involved, particularly in relation to dinoflagellate host.

The Dinophysoid dinoflagellate groups (class Dinophyceae, order Dinophysiales) contain the genera *Amphisolenia*, *Triposolenia*, *Citharistes*, *Histioneis*, *Parahistioneis*, and *Ornithocercus* which are known to have cyanobionts. Of these genera, the first two contain intracellular cyanobionts, and the others contain extracellular cyanobionts^11–15^. Early studies on the dinophysoid dinoflagellates have exclusively focused on the morphological features of cyanobionts, such as shape, size, and thylakoid arrangement based on ultrastructural observations^15–18^. Later, molecular techniques were applied to the dinophysoid cyanobionts revealing their genetic diversity^19^. These studies have been conducted exclusively in the tropical and subtropical Pacific, Atlantic, and Indian Oceans, because cyanobiont-bearing dinoflagellates are commonly found in the low-nutrient conditions of the open ocean^12,15–17,19–21^. In contrast, free-living picocyanobacteria, such as *Synechococcus* and *Prochlorococcus*, are widely distributed, including in temperate waters undergoing seasonal changes in water temperature. As such, they can be subdivided into cold/warm types associated with variations in water temperature^22^.

The Pohang coastal area and the Jeju Strait off the coast of Korea are located in temperate waters. These regions undergo large seasonal variations in water temperature and salinity^23–25^, owing to inputs from the Tsushima Warm Current and the Yangtze River^26–28^. Therefore, the possible occurrence of dinophysoid dinoflagellates in such temperate coastal waters could raise several questions regarding their cyanobionts. For example, do the dinophysoid dinoflagellates acquire new cyanobionts seasonally from the surrounding waters? Do dinoflagellate hosts maintain constant cyanobiont compositions irrespective of variations in water temperature and salinity? To address these questions, a total of 138 individual *Ornithocercus* cells were isolated from 13 surveys over four seasons (March (winter), June (spring), September (summer), and November and December (autumn)) in three years. To identify the genetic type of *Ornithocercus* cyanobionts present, the cyanobacterial 16S rDNA (V3–V4 regions) sequences of whole cyanobionts from individual host cells were verified using the Illumina MiSeq sequencing platform.

## Results

### Microscopic observations of the cyanobionts

**Figure 1:**
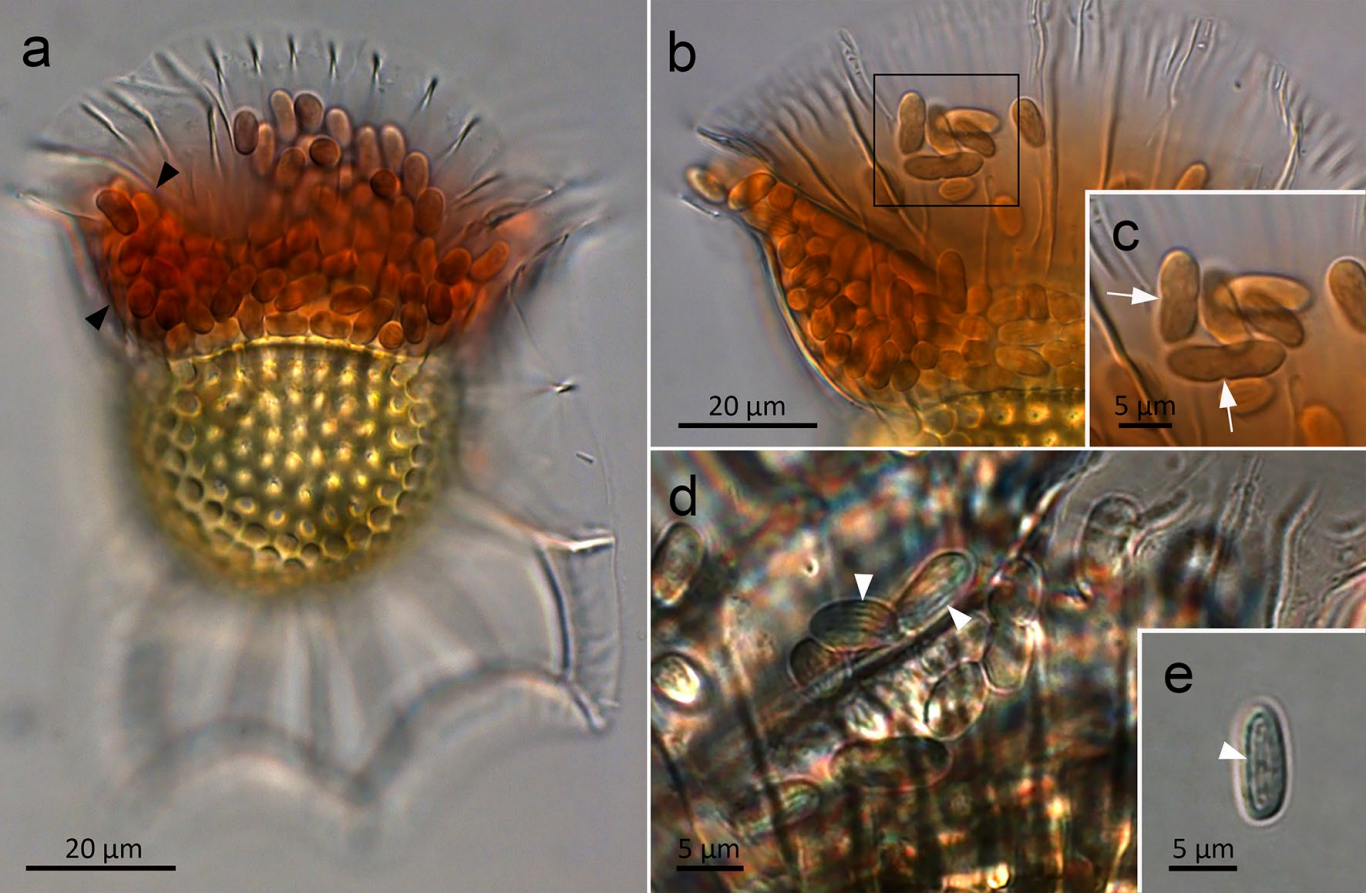
Micrographs of live cyanobacterial symbionts (cyanobionts)-dinoflagellate *Ornithocercus* host consortium. (**a**) *O. magnificus* with numerous cyanobionts present in the upper and lower girdle lists (black arrowheads) of the cingulum termed the symbiotic chamber. (**b**) *O. steinii* with numerous cyanobionts inhabiting the symbiotic chamber. (**c**) Enlargement of the area indicated by the box in 1b showing two cyanobionts that are being divided by binary transverse fission (white arrows). (**d**) Thylakoid membranes (white arrowheads) of *O. steinii* cyanobionts. (**e**) An *O. steinii* cyanobiont that has escaped from the same *O. steinii* host as shown in 1d, showing thylakoids in concentric layers (white arrowhead).

Most *Ornithocercus* specimens collected from field samples contained cyanobionts that inhabit the space between the upper and lower girdle lists of the cingulum called the symbiotic chamber (Fig. 1). The cyanobionts were rod-shaped, measuring on average 8.38 µm in length and 4.06 µm in width (n = 30) and were reddish brown in color under a light microscope, with bright orange fluorescence emitted under green-light excitation. Spherical shaped cyanobionts smaller than the rod-shaped cyanobionts were rarely observed. Among these cyanobionts, cells undergoing transverse binary fission were often observed (Fig. 1b,c). The thylakoids of the cyanobionts consisted of concentric layers (Fig. 1e).

### Screening for high-throughput sequencing runs of cyanobionts

**Figure 2:**
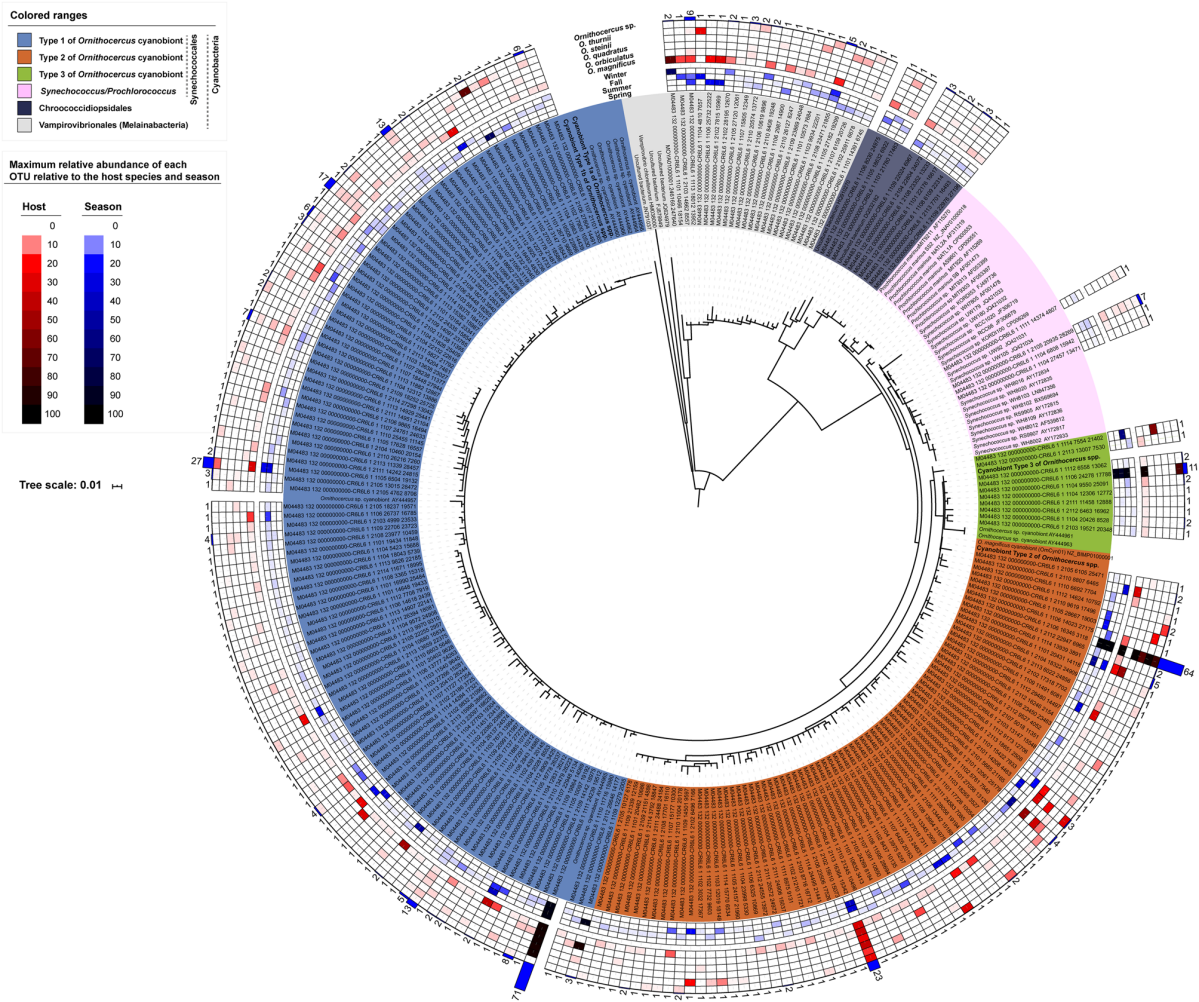
A circular phylogenic tree with heatmaps of the cyanobacterial operational taxonomic units (OTUs) obtained from *Ornithocercus* symbionts. The tree was constructed based on the V3–V4 regions of the cyanobacterial 16S rDNA gene using the neighbor-joining method. The two heatmaps represent the maximum relative abundance of each OTU to host species (outer) and season (inner). The color ranges of the heatmaps represent the relative percentage for each OTU. The outermost bars indicate the number of *Ornithocercus* cells associated with each OTU. The phylogenetic tree and heatmaps were constructed using the online tool, Interactive Tree of Life (iTOL Version 5.7).

A total of 4,422,006 high quality sequences of the cyanobacterial 16S rDNA (V3-V4 regions) were obtained from 138 individual *Ornithocercus* cells, with on average 32,044 reads per individual cell assigned. For each sample, the operational taxonomic units (OTUs) with sequences ≥ 1% of the total reads were selected. These sequences were 98%–100% similar to those of three cyanobacteria order Synchococcales, Chroococcidiopsidales, and Vampirovibrionales and were clustered into 229 OTUs (Fig. 2). The most common order in the dataset was Synchococcales, accounting for 86% (197 OTUs) of the total OTUs, followed by Vampirovibrionales (Melainabacteria) at 9.6% (22 OTUs) and Chroococcidiopsidales at 4.4% (10 OTUs). Based on the MiSeq data, *Ornithocercus* cyanobionts belonging to the Synchococcales could be divided into three major genetic types, i.e., Type 1, Type 2, and Type 3, accounting for 111 (48.5%), 74 (32.3%), and 8 (3.5%) OTUs, respectively. All three cyanobiont types showed no significant pattern of occurrence in relation to host species and seasonal change.

### Phylogenetic analyses of Ornithocercus cyanobionts

**Figure 3:**
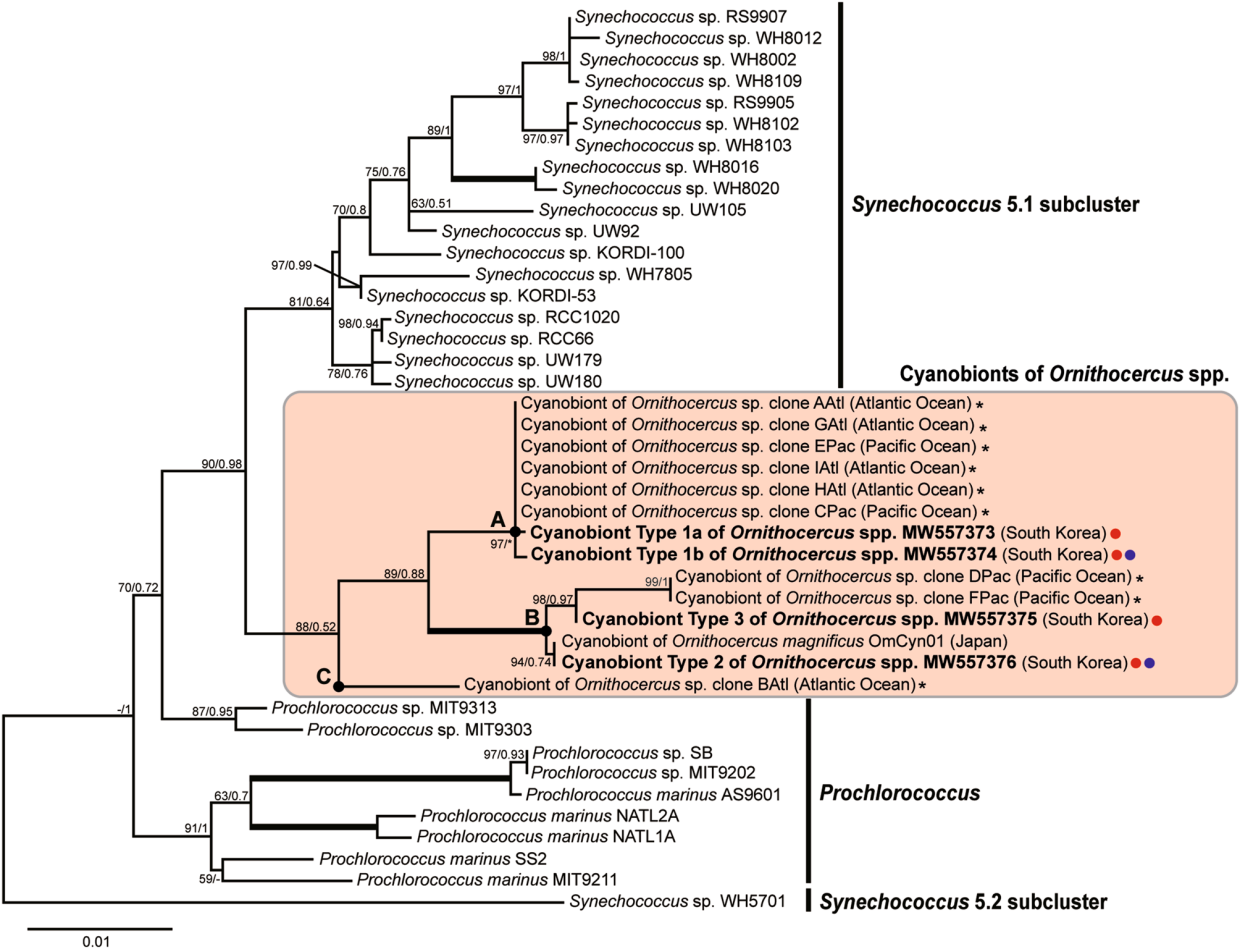
The Maximum Likelihood (ML) phylogeny of cyanobacterial symbionts (cyanobionts) from a dinoflagellate *Ornithocercus* host and marine picocyanobacteria based on combined data of the cyanobacterial partial 16S rDNA-entire ITS gene. Bold text indicates the *Ornithocercus* cyanobiont sequence obtained in this study. Red and blue circles represent amplified sequences from single-host DNA and single-cyanobiont DNA, respectively. The ML tree was inferred using IQ-TREE. Branch support was obtained from bootstrap values and a Bayesian posterior probability. A thick branch denotes a strongly supported bootstrap value of 100% and the highest posterior probability (1). The hyphen represents the unmatched tree topology with the Bayesian tree. Letters on branches (**A**–**C**) refer to clades of *Ornithocercus* cyanobionts. Asterisks represent the taxa of which only short 16S rDNA sequences (V3–V4 regions) were analyzed. The letters in parentheses indicate the geographic origins of the *Ornithocercus* cyanobionts. The scale bar represents the substitution per site.

*Ornithocercus* cyanobionts formed a monophyletic clade and were placed as a sister group to *Synechococcus* subcluster 5.1 within the picocyanobacterial groups. Phylogenetic analyses based on the cyanobacterial partial 16S rDNA-internal transcribed spacer (ITS) gene revealed that *Ornithocercus* cyanobionts comprised three distinct clades (A, B, and C): clade A contained six clone sequences (V3–V4 region of 16S rDNA) of *Ornithocercus* cyanobionts isolated from the Atlantic and Pacific Oceans and Type 1 sequences obtained in this study; clade B contained Type 2 sequences, which were 100% identical to a sequence (OmCyn01) isolated from Japan, and Type 3 sequences newly discovered in this study; and clade C contained one sequence of *Ornithocercus* cyanobionts isolated from the Atlantic Ocean (red circles in Fig. 3). Type 1 sequences were further subdivided into Type 1a and 1b. These two subtypes displayed only one sequence difference in the 16S rDNA, However, there were distinct differences in 25 nucleotides where one gap was observed in the ITS sequences. Overall, while the partial 16S rDNA genes (1140 bp) of the *Ornithocercus* cyanobionts were quite conservative owing to low sequence variation between cyanobionts, results for the ITS genes indicated that they were highly variable in terms of both length and sequence (Table 1, Table S4).

**Table 1:** A genetic p-distance matrix of the partial 16S rDNA and entire ITS gene in three genetic types of *Ornithocercus* cyanobionts sampled in this study.

*Ornithocercus* cyanobionts	Partial 16S rDNA					Entire ITS				
	Size (bp)	Type 1A	Type 1B	Type 2	Type 3	Size (bp)	Type 1A	Type 1B	Type 2	Type 3
Type 1a	1140	–	–	–	–	754	–	–	–	–
Type 1b	1140	0.1	–	–	–	753	3.3	–	–	–
Type 2	1140	1.2	1.1	–	–	697	18.1	17.6	–	–
Type 3	1140	1.3	1.2	0.3	–	708	17.9	17.6	4.0	–

### Hydrographical characteristics of the study area

**Figure 4:**
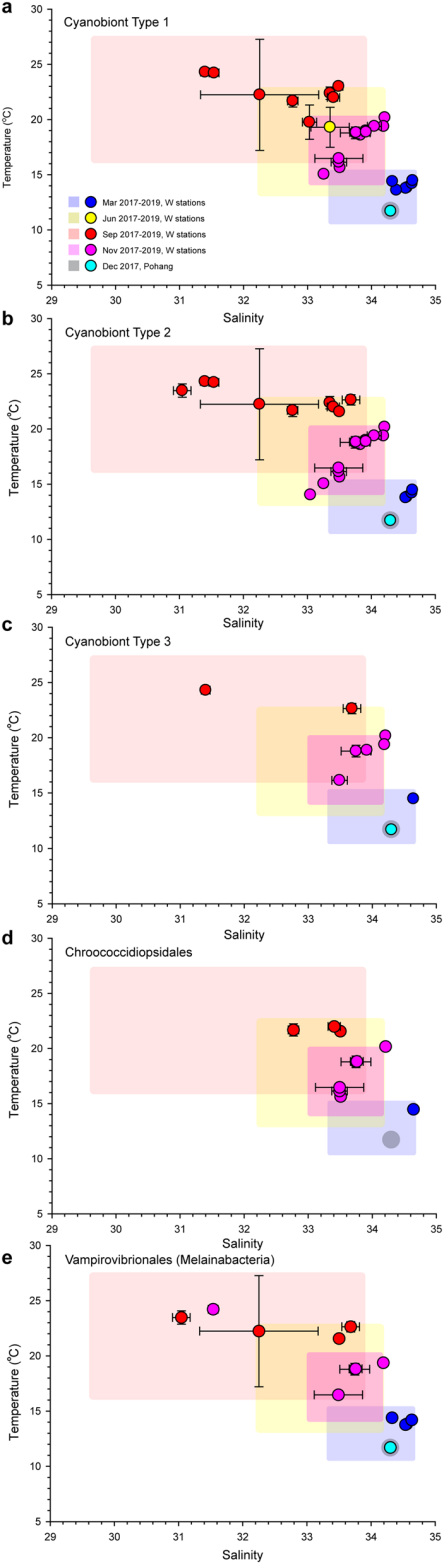
Temperature and salinity levels at stations where *Ornithocercus* cyanobionts were detected from isolated host cells sampled during different seasons (March, June, September, and November 2017–2019). (**a**–**c**) Stations where three genetic types of Synechococcales cyanobionts were detected; (**d**,**e**) Stations where Chroococcidiopsidales and Vampirovibrionales cyanobionts were detected. Square areas show the temperature-salinity range measured at the sampling stations. Error bars represent the standard deviations (SD) for temperature and salinity between surface and 30 m depths.

Seawater temperature changes seasonally in temperate waters. In the study area, seasonal mean temperatures ranged from 11.1 to 25.2 °C, and minimum and maximum temperatures were 10.5 °C and 27.6 °C, respectively (Fig. 4). Vertical temperature differences (surface to 30 m in depth) in June and September were 4 °C and 2.7 °C, respectively, which were relatively large compared to those in November and March (Supplementary Table S3). Surface seawater temperature tended to increase from station W1 toward station W9 along the transect throughout the year. The seasonal mean for salinity ranged from 31.3 to 34.5 (Supplementary Table S3). Considering only March, June and November, the salinity range variation was slightly narrow, with a minimum of 32.2 and a maximum of 34.2 (Fig. 4). In September, however, the salinity variation was very large, from a minimum of 29.6 to a maximum of 33.9, most likely a result of large inputs from the Yangtze River during this season. The seawater temperature and salinity at the Pohang station in December was 11.7 °C and 34.3, respectively (Fig. 4).

### Cyanobionts diversity and abundance relative to seasons and host species

**Figure 5:**
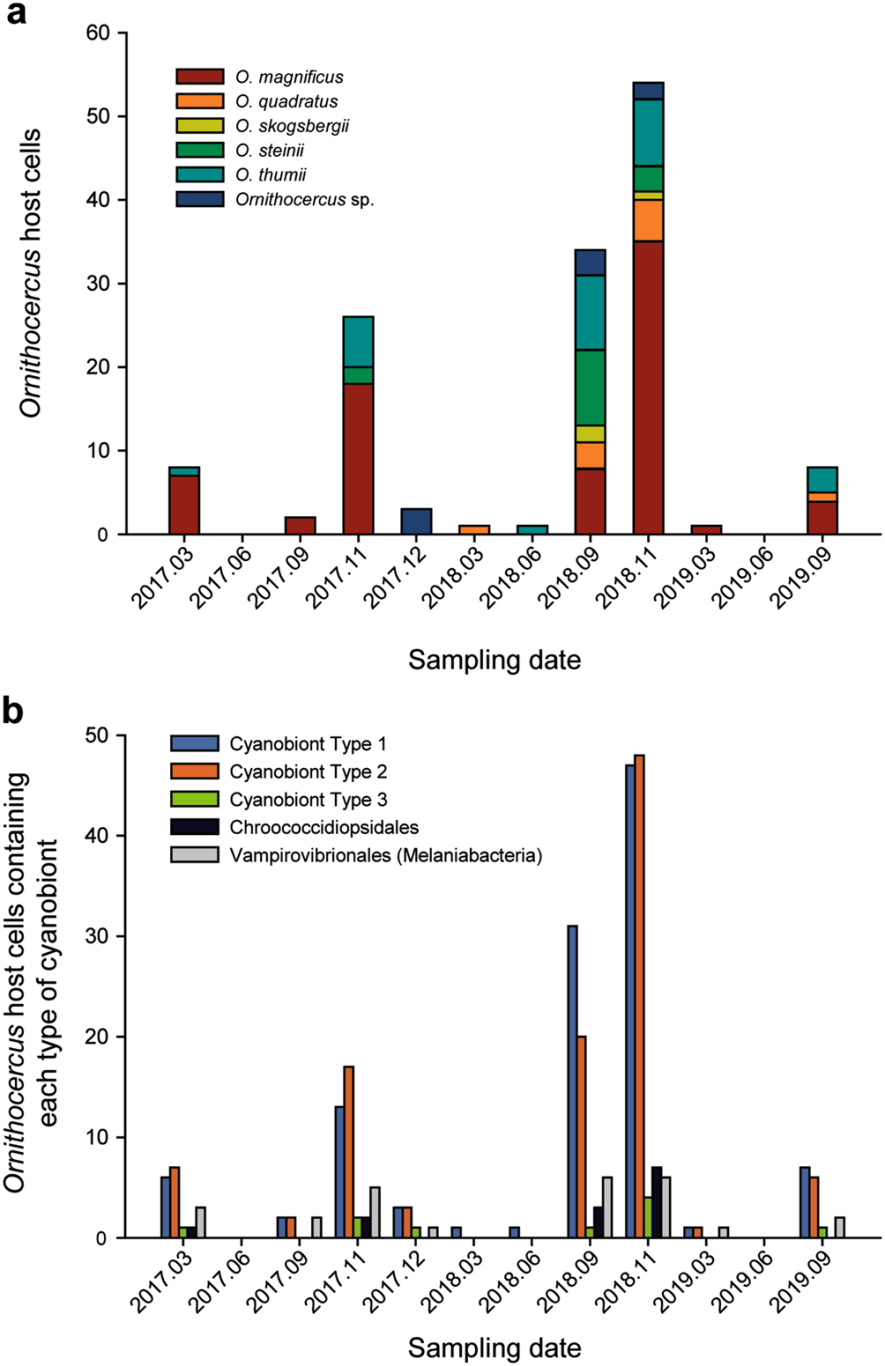
The number of individual cyanobiont-bearing *Ornithocercus* cells in temperate waters. (**a**) Seasonal changes in *Ornithocercus* populations showing the five host species and unidentified species encountered in the study areas. (**b**) The number of *Ornithocercus* hosts showing seasonal variations in symbiont types.

**Figure 6:**
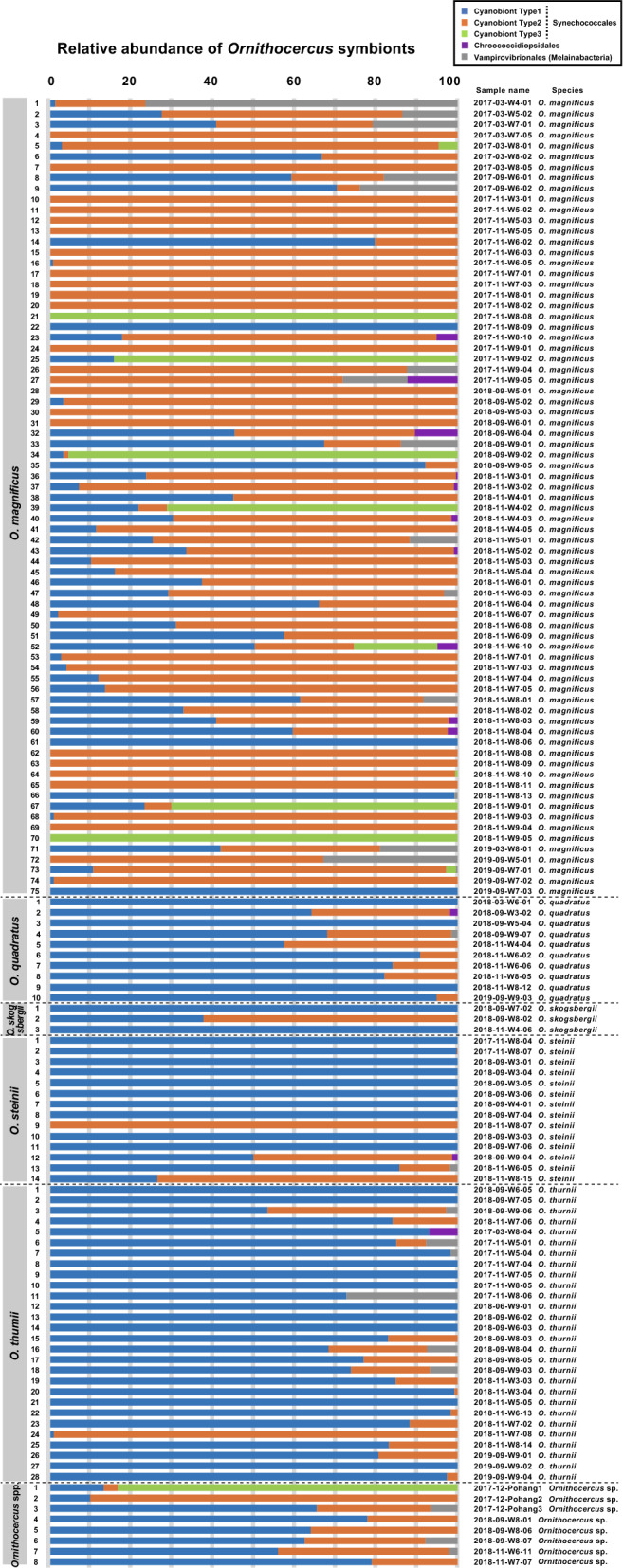
The relative abundance of each symbiont to single *Ornithocercus* cells. Note that it is listed by host species.

Individually isolated host cells were classified to species as follows: 74 *O. magnificus*, 16 *O. orbiculatus*, 10 *O. quadratus*, 5 *O. steinii*, 24 *O. thumii*, and 8 unidentified *Ornithocercus* species. *O. magnificus* was the most abundant host species in the study area. It was the most abundant species in November (autumn), but was rarely found in June (spring) (Fig. 5a). Type 1 and 2 cyanobionts occurred together in most samples except in March. and June 2018 (Fig. 5b) when, the temperature ranged from 11.7 to 24.3 °C and salinity ranged from 31.0 to 34.6 (Fig. 4a,b). In contrast, Type 3 cyanobionts were observed mostly in autumn (November and December) when the temperature and salinity ranges were 11.7–22.6 °C and 33.5–34.3, respectively (except for one cell which was found at a low salinity of 31.4) (Fig. 4c). Type 1 and 2 Synechococcales cyanobionts were observed in all host species examined (Fig. 6). In particular, a large portion of the Type 2 cyanobionts (66%) observed were found in *O. magnificus* and were the predominant cyanobiont type in this host (Fig. 6). While Type 1 cyanobionts were occasionally observed at low abundances, specifically in *O. magnificus* cells, they were found in high abundances in *O. orbiculatus*, *O. quadratus, O. steinii*, *O. thumii*, and the unidentified *Ornithocercus* cells (except for one *O. orbiculatus* and one *O. thumii* cell in which Type 2 dominated) (Fig. 6). Type 3 cyanobionts were observed exclusively in a small number of *O. magnificus* cells, except for one unidentified *Ornithocercus* cell (outer heatmap in Fig. 2 and Fig. 6). Over half (59.4%) of the host species contained different types of Synechococcales cyanobionts simultaneously, most of which were a combination of Types 1 and 2 (89%). The combinations of Types 1 and 3 (1.2%) or Types of 2 and 3 (1.2%) were rarely observed. In seven specimens (8.5%), all three types were detected at the same time (Fig. 6). Type 1 cyanobionts were found in 81.2% of the *Ornithocercus* populations, followed by Type 2 at 75.4%, and Type 3 at 7.2% (Fig. 6). In addition to the Type 1, 2, and 3 Synechococcales cyanobionts, low abundances of cyanobacterial symbionts, Chroococcidiopsidales (9.4%) and Vampirovibrionales (18.8%) (Melainabacteria), were detected in some cells sampled in September (summer), November, and December (Figs. 2, 6).

### Phylogenetic clustering of cyanobionts from dinophysoid hosts of different origin

**Figure 7:**
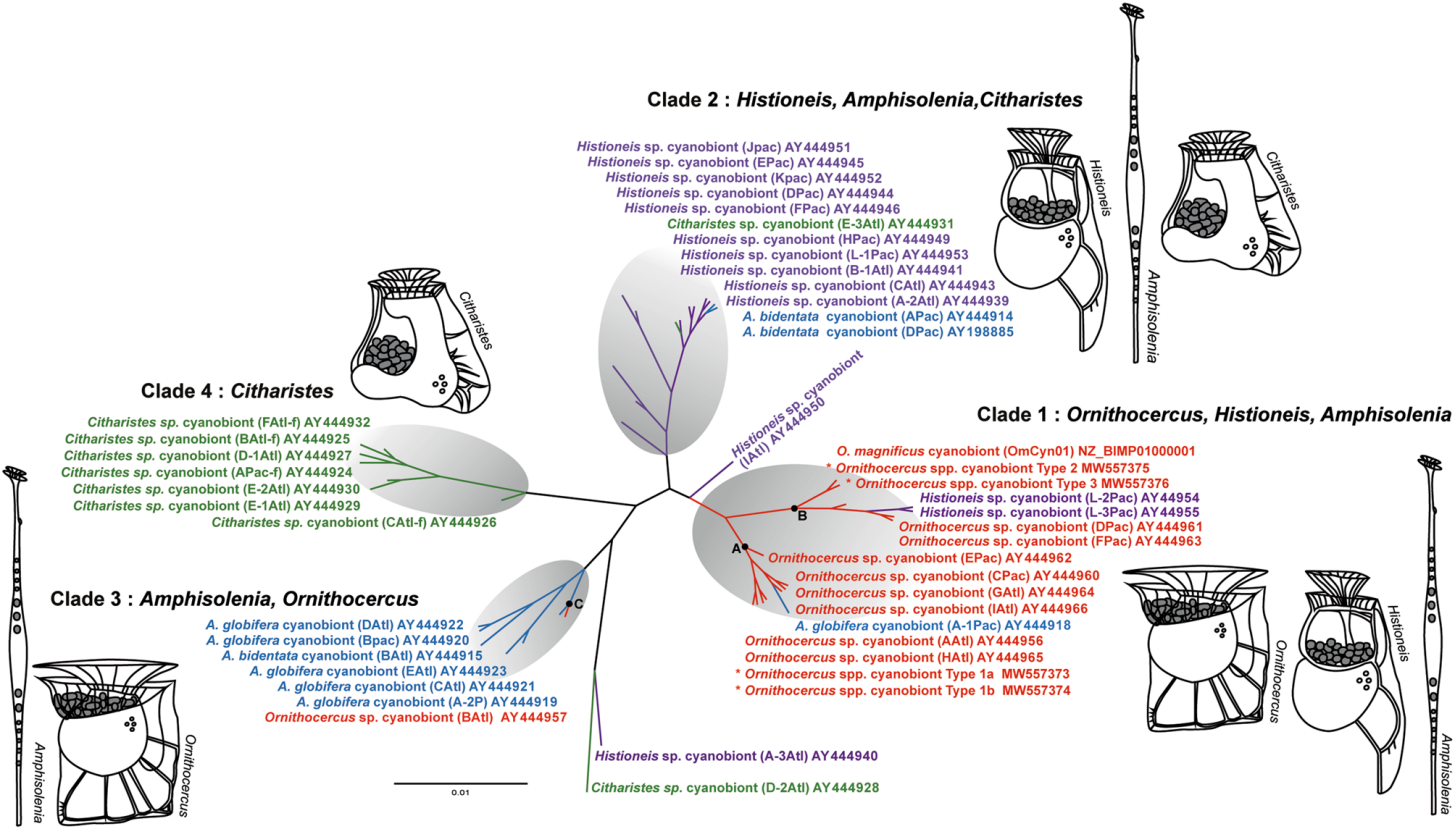
Phylogenetic clustering of dinoflagellate hosting-cyanobionts. A maximum Likelihood tree based on the V3–V4 regions of the cyanobacterial 16S rDNA gene was inferred using IQ-TREE under the TN + F + R2 model. Each host genus with cyanobionts (grey) is illustrated. Different colors of text and lines represent each host genus. Capital letters indicate the genetic clade of *Ornithocercus* mentioned in Fig. 3.

Based on the 16S rDNA sequences (V3–V4 regions) of the dinophysoid dinoflagellate cyanobionts that are registered in GenBank (*Amphisolenia*, *Citharistes*, *Histioneis*, and *Ornithocercus*), including sequences obtained from the present study, phylogenic relationships (among these cyanobionts) were divided into four clades. In addition, our results indicated a moderate host-cyanobiont relationship to some extent at the genus level (Fig. 7). All but one *Ornithocercus* cyanobionts (AY444957) clustered together with one *Amphisolenia* cyanobiont (AY444918) and two *Histioneis* cyanobionts (AY44954 and AY44955) (clade 1; Fig. 7). The *Histioneis* and *Amphisolenia* cyanobionts formed clades 2 and 3, respectively, although each clade included one to three cyanobionts from different host genera. Except for two *Citharistes* cyanobionts (AY444931 and AY444928) placed in different positions, all the other *Citharistes* cyanobionts clustered into clade 4, which comprised only one dinophysoid host species. In addition, neither of the two *Histioneis* cyanobionts (AY444950 and AY444940) nor a *Citharistes* cyanobiont (AY444928) were included in any clade, and the latter two were closer to *Prochlorococcus* (with a similarity of 97.4 to 98.7%) than to any dinophysoid cyanobionts.

## Discussion

Dinophysoid dinoflagellates are among the rarest of species within planktonic communities, and long laboratory cultures of them have not been established. Nonetheless, they have received much attention because they harbor unique cyanobacterial symbionts (i.e., cyanobionts) inside or outside the cell and so, are useful when studying several aspects of the cyanobiont-dinoflagellate host consortia. In the present study, genetic analysis of cyanobionts from the dinoflagellate host *Ornithocercus* isolated from samples obtained over four seasons from temperate waters revealed that dinoflagellate hosts harbor moderately host-specific and consistent types of cyanobionts throughout the year.

In general, the ITS gene can successfully distinguish closely related cyanobacterial species strains^29–31^. Indeed, the present study has shown that Type 1 Synechococcales cyanobionts can be further subdivided into Type 1a and 1b when based on ITS sequences, revealing the presence of four genetic types of *Ornithocercus* cyanobionts (Supplemental Fig. S2a,b). In addition, the phylogenetic position of the *Ornithocercus* cyanobiont (OmCyn) was better represented when based on the ITS sequence than on the 16S rDNA sequence^32^. Despite an indication of the presence of at least four distinguishable genetic types of *Ornithocercus* cyanobionts based on the ITS sequences, they were treated as three types in this study because all samples were processed based on the V3-V4 regions of the cyanobacterial 16S rDNA gene.

The Types 1 and 2 Synechococcales cyanobionts found in this studywere very similar to the *Ornithocercus* cyanobionts found in the Atlantic and Pacific Oceans and were identical to those found in coastal waters of Japan^19,32^. In this study, most of the cyanobionts associated with the *Ornithocercus* cells were rod-shaped with an orange fluorescence, as previously reported^20,32^, and were, on average 8 µm in length and 4 µm in width (Supplemental Fig. S2a,b). Unfortunately, the cell shapes of Type 3 cyanobionts could not be identified in this study; however, considering the observation that most cyanobionts are rod-shaped, they are likely to be rod-shaped as well. Alternatively, Type 3 cyanobionts may have a different shape in terms of morphology and ultrastructure compared to Types 1 and 2. Previous ultrastructural studies have reported the several different morphotypes of *Ornithocercus* cyanobionts such as rod/ellipsoid or spherical shapes, and concentric, central/peripheral, or transverse thylakoids, and these cyanobionts were much smaller (1–3.5 µm) than those previously cited^12,15,16,20^. Small-sized cyanobionts may have been underestimated by the direction of the TEM sections performed in the previous studies or simply overlooked in this study. It is interesting to note the huge size difference between the very large Synechococcales cyanobionts of *Ornithocercus* compared to the tiny free-living *Synechococcus* and *Prochlorococcus*, which have a size of 0.8 to 1.5 µm. Similarly, the endosymbiotic cyanobacteria of the amoeba *Paulinella chromatophora* are relatively large compared to their free-living relatives, measuring approximately 15–20 µm and 3.5–4 µm in length and diameter, respectively^33^.The reason for this is currently unknown, but the cyanobacteria in symbiotic relationships may have attained such large sizes as a result of physiological or genetic interactions (e.g. nutrient transfer, gene transfer, gene loss) with their host. Unlike the free-living picocyanobacterial community, whose distributions changes dynamically with fluctuations in water temperature and nutrient levels^22^, it is noteworthy that the community composition and distribution of *Ornithocercus* cyanobionts belonging to the Synechococcales are not significantly affected by seasonal changes in water temperature. In this study, all three types of Synechococcales cyanobionts were found to occur in a wide range of water temperatures from 11 to 24 °C. Instead, their distribution seemed to be closely associated with variation in salinity. Types 1 and 2 occurred in a salinity range of 31–34.6, while the distribution of Type 3 was mainly confined to a relatively narrow range of 33.5–34.6 (except for one cell detected at low abundance in a host specimen with Type 3) (Fig. 4c and 2019-09-W7-01 in Fig. 6). This suggests that Types 1 and 2are a euryhaline species and Type 3 is a stenohaline species. The picocyanobacterium *Prochlorococcus* has been known to be advected from warm, oligotrophic open oceans to temperate waters by currents^22^. Similarly, it seems likely that already established cyanobiont-*Ornithocercus* host consortia from the tropical region were moved to temperate regions such as the current study area by the Tsushima Warm Current rather than newly forming the consortia by either acquiring cyanobionts or choosing a host upon arrival. If this is true, then, why do the three types of Synechococcales cyanobionts display different patterns of distribution relative to salinity? Perhaps, this is because, while Types 1 and 2 can tolerate variation in salinity and readily adapt to temperate waters, Type 3 may be intolerant to such variations in salinity and thus fail to adapt to the new environment. Further study is needed to address the ecophysiological adaptations of the Synechococcales cyanobionts relative to variations in salinity. Unfortunately, nutrient concentrations in the study area were not measured, but this area is known to have nutrient levels higher than oligotrophic oceans, but lower than coastal areas^34^. Given that cyanobionts similar to Types 1 and 2 have been detected across a wide range of aquatic environments, from oligotrophic equatorial, tropical, and subtropical oceans to even coastal areas^19,32^, it is unlikely that cyanobiont populations are significantly affected by nutrients levels.

In the cyanobiont-dinophysoid host consortia, it remains unknown whether the dinophysoid host actively searches for and selects specific cyanobiont(s) as the symbiotic partner or whether cyanobiont(s) choose each preferred host. Nonetheless, results from this study indicated the presence of a certain degree of host (or cyanobiont) specificity in cyanobionts (or host) among *Ornithocercus* species as well as among dinophysoid species. Given that Types 1 and 2 were detected in 81.2% and 75.4%, respectively, of the 138 *Ornithocercus* specimens isolated in the present study, it seems likely that both types are dominant Synechococcales cyanobionts. Therefore, it is likely that Foster et al.^19^ also detected large numbers of Synechococcales cyanobionts, which are genetically similar to Type 1 cyanobionts, in most of the *Ornithocercus* hosts isolated from subtropical and tropical regions. More importantly, the present study found that more than half of the *Ornithocercus* specimens (59.4%) sampled contain multiple types of Synechococcales cyanobionts simultaneously, most of which were a combination of Types 1 and 2 (89%). The relative dominance of these types within each specimen, however, tended to be dependent on the *Ornithocercus* species. While Type 2 tended to be predominant in *O. magnificus*, Type 1 was predominant in other *Ornithocercus* species (i.e., *O. orbiculatus*, *O. quadratus*, *O. steinii*, and *O. thumii*) except in *O. magnificus*. Very recently, the new finding of a cyanobiont (OmCyn), which appears to be the same as the Type 2 in the present study, by a Japanese research group^32^ must have resulted from the use of a single *O. magnificus* specimen, thereby making it easy to detect. It should also be noted that Type 3 was observed exclusively in *O. magnificus*, except for one unidentified *Ornithocercus* cell. The relative abundance values of the cyanobiont types in each *Ornithocercus* cell may have been affected to a greater or lesser extent by amplification bias in the PCR procedure. However, this did not significantly affect the finding that this cyanobiont type was host species dependent. Furthermore, phylogenetic analysis of the Synechococcales cyanobionts from dinophysoid species (i.e., *Amphisolenia*, *Citharistes*, *Histioneis*, and *Ornithocercus*) produced four distinct clades, with each clade consisting of specific Synechococcales cyanobionts, except for a few outliers (Fig. 7). We frequently observed cyanobionts in the process of cell division in the host cingulum and their transmission vertically along with the division of the host cell (Supplemental Fig. S2c), as reported in previous studies^9,16^. In terms of the symbiont transmission mode, host dependence on vertically transmitted symbionts tends to be higher than that of horizontally transmitted symbionts^35^. In addition, according to size fractioned global cyanobacterial sequences based on the Tara Oceans metagenomics data, *Ornithocercus* cyanobiont sequences have been predominantly retrieved in the host-size category (20–180 µm) rather than in the cyanobiont size category (0.8–20 µm)^32^, This suggests that the life history of cyanobionts is obligately dependent on their host compared to the free-living life stage. Even the reduced genome of the *Ornithocercus* cyanobiont, with significantly reduced metabolic capacities for photosynthesis and nitrogen fixation, likely explains why cyanobionts cannot live on their own in the free-living phase^32^. Such a highly dependent host-cyanobiont relationship may have allowed them to have either host specificity or cyanobiont specificity. Taken together, the results from this study suggest that host (and/or Synechococcales cyanobionts) niche separation is present among *Ornithocercus* species as well as dinophysoid species. In addition to the Synechococcales cyanobionts, this study identified OTU sequences affiliated with the Vampirovibrionales (Melainabacteria) and Chroococcidiopsidales in some *Ornithocercus* cells. Both cyanobacterial groups have frequently been found in extreme and dynamic environments such as marine sediment, rocks, grassland soil, and guts^36–38^. It was hitherto unknown that *Ornithocercus* species are a habitat for these bacterial groups. The Melainabacteria group have been found to lack the photosynthetic function^36–38^, but overall, the physiological characteristics of these two bacterial groups are still unknown. Further study is needed to better understand relationship dynamics between these cyanobionts and their host. In addition, more research is required to ascertain the prevalence of those bacterial groups depending on the dinophysoid host or geographic region.

## Methods

### Study area and sample collection

This study was conducted along the coast of the East sea (1 station, Pohang) and the coast of the South Sea of Korea (9 stations between Wando and Jeju) seasonally affected by inflow from the Jeju Warm Current, a branch of the Tsushima Warm Current (Supplementary Fig. S1). The Pohang sample was collected from the coastal water using a 0.3 m diameter plankton net with a 20 µm mesh in December 2017. Except for the Pohang sample, all samples were vertically hauled through the water column from a depth of 30 m to the surface using a 0.6 m diameter bong net with a 20 µm mesh from a cruise ship every March, June, September, and November for 3 years from 2017 to 2019. Fifty mL of the highly concentrated plankton samples were immediately fixed with 2% neutral Lugol’s solution in a polyethylene bottle and wrapped in foil later for microscopic observation. The remaining sample was poured into a 10 L bucket containing seawater for live-cell observations in the laboratory. Oceanographic parameters were measured from discrete depths using a CTD profiler (SBE 19plus V2, Sea-Bird Electronics, USA) mounted on a rosette sampler at each station.

### Single-cell isolation for DNA extraction and light microscopy

From the fixed and live plankton samples, 138 single *Ornithocercus* host cells with cyanobionts were isolated under an inverted microscope (Axio Vert.A1, ZEISS, Germany). The host cells were first photographed for host species identification and observation of their symbionts. Light micrographs of the fixed host cells were obtained at magnifications of 200 × and 400 × using the inverted microscope equipped with a full HD mini box camera (MediCAM-Z, Comart System, Korea). After obtaining the micrographs, individual host cells with cyanobionts (at least five cells per station) were drawn using Pasteur glass pipettes, gently washed five times in sterile seawater, and put into separate 0.2 mL PCR tubes containing 50 µL of 10% Chelex (Bio-Rad, USA). In addition, only one cyanobiont cell from a live-host cell was isolated and processed in the same manner as described above in order to obtain a single symbiont DNA. In contrast, the live host cells were isolated using glass pipettes, placed on a microscope slide, and photographed at magnifications of 630 × and 1000 × using an AxioCam HRc (Carl Zeiss Inc., Germany) coupled to a Zeiss Axio Imager A2 microscope equipped with differential interference contrast optics. Subsequently, the individual live cells were carefully taken back from the slide and then treated in the same way as above. The PCR tubes were boiled at 95 °C for 1 h and then centrifuged at 13,000 rpm for 5 min. The supernatant (30 µL), which contained the genomic DNAs of single host cells and its extracellular symbionts, was harvested.

### Amplification of cyanobacterial 16S rDNA for sequencing using an MiSeq platform

Extracted DNAs were amplified using the V3-V4 hypervariable regions of the 16S rDNA of cyanobionts. The libraries of the 16S rDNA were prepared for Illumina MiSeq sequencing using two modified primer pairs with ligated Illumina overhang adapter sequences on both forward and reverse primers (Pro314F and Pro805R/CYA359R and CAY781R in Supplementary Table S1)^39–42^. General procedures for PCR amplification, clean up, and indexing PCR for MiSeq sequencing followed the instructions described in the MiSeq manual^43^. PCR was performed in 25 µL reaction mixtures, which included 3 µL of genomic DNA, 0.7 µLeach of the 10 µM primers, 0.5 µL of the 10 mM dNTPs, and 0.6 units of Diastar Taq polymerase (Solgent, Korea). The first-round PCR consisted of a 10 min denaturation step at 94 °C, followed by 40 cycles of 30 s at 94 °C, 30 s at 53 °C, and 50 s at 72 °C. Each amplicon was then amplified using an Illumina universal index primer i5 and i7 for multiple samples to be pooled and sequenced in a single run. In nested PCR reactions, 3 µL of the PCR product from the first-round PCR analysis was used as a template, and the PCR was run under conditions as described above, except for 35 instead of 40 cycles and an annealing temperature of 60 °C instead of 53 °C. The resultant products were purified using a LaboPass PCR purification kit (Cosmogenetech, Korea), normalized by a Qubit 3 fluorometer (ThermoFisher, USA), and pooled into a 1 mL tube. Amplicon sequencing was performed by Chunlab, Inc. (Korea) using an Illumina MiSeq platform.

### Data analyses

Reads from the MiSeq sequencing were analyzed using the program Mothur (Version 1.44.2)^44^ as suggested by the MiSeq standard operating procedures (http://www.mothur.org/wiki/MiSeq SOP)^45^. After assembling paired-end sequences, contigs with ambiguous bases (N) over two, shorter than 400 bp or longer than 525 bp, and homopolymers greater than nine were removed. The EzBioCloud 16S database was used as a reference database for alignment and classification in the Mothur program (https://www.ezbiocloud.net/resources/16s_download). After alignment, sequences that were likely due to PCR errors were removed using the ‘pre.cluster’ command, which permits up to four differences between sequences; then, chimeric sequences were also removed using the ‘chimera.vsearch’ command. The remaining sequences were assigned to operational taxonomic units (OTUs) using a 99% sequence identity threshold and then classified using the naïve Bayesian classifier with a bootstrap cut-off of 80%^46^. Finally, OTUs classified as the Cyanobacteria phylum were selected for further analyses.

### Amplification of partial 16S rDNA and the ITS region of a cyanobiont

To verify additional genetic information of the cyanobionts, DNA samples from a single host cells, that predominantly contained one type of symbiont, were selected based on the results of the MiSeq sequencing data. Various types were defined as follows: (Type 1A) 2018-09-W3-03 (single-host DNA), (Type 1B) 2018-11-W5-05 (single-host DNA) and 2019-11-W9-01 (single-cyanobiont DNA), (Type 2) 2018-11-W9-04 (single-host DNA) and 2019-11-W4-02 (single-cyanobiont DNA), and (Type 3) 2018-11-W9-05 (single-host DNA). Additionally, individual cyanobionts were isolated from the single host cells (sampled in November 2019), and single-cell DNA was extracted following the method described by Kim and Park (2019)^47^. DNA samples of single hosts and single cyanobionts were subjected to PCR amplification of the cyanobacterial 16S rDNA (partial)-entire ITS region. The amplification of each gene was necessarily accompanied by a nested PCR assay because the amount of single-cell DNA was not sufficient to be detected in one PCR assay. All amplifications were performed using 3 µL of genomic DNA and primer sets (Supplementary Table S1) under the following PCR conditions: 10 min at 94 °C, followed by 40 cycles of 30 s at 94 °C, 30 s at 55 °C, and 2 min at 72 °C. The nested PCR conditions were the same as those used in the first-round amplification, except for 35 instead of 40 cycles and an annealing temperature of 57 °C instead of 55 °C. Amplified DNA fragments were purified using a LaboPass PCR purification kit (Cosmogenetech, Korea), and sequencing was performed by Applied Biosystems (Cosmogenetech, Korea).

### Phylogeny

First, the phylogenetic tree for the representative sequences of OTUs were constructed using the ARB program (Fig. 2). Each sequence with length of 403 bps was added using “add species to existing tree” with ARB to a reference tree which was composed of sequences from the EzBioCloud, sequences retrieved from the GenBank and near full-length sequences obtained in this study. The phylogenetic tree for the representative sequences of OTUs were constructed by neighbor-joining method using the Geneious (Version 7.1.3) program. The length of the alignment for the tree construction was 385bps.The results were presented as a phylogenetic tree and heatmaps using the online tool, Interactive Tree of Life (iTOL Version 5.7, https://itol.embl.de)^48^. Second, two phylogenetic trees based on the data set of both cyanobacterial 16S rDNA-ITS genes (Fig. 3) and the V3–V4 regions of the 16S rDNA gene in dinoflagellate hosting-cyanobionts (Fig. 7) were inferred using two model-based methods, Maximum Likelihood and Bayesian inference, respectively. For the phylogeny of the cyanobacterial 16S rDNA-ITS genes, the alignment data were generated by combining the cyanobacterial 16S rDNA and the ITS gene sequences. The obtained sequences were primarily aligned and edited with previously known sequences in the database obtained from GenBank (Supplementary Table S2). Alignment was performed by eye using Genetic Data Environment (GDE 2.4), and positions that could not be aligned unambiguously were omitted from analysis. A total of 1568 and 321 unambiguous sites were analyzed for the cyanobacterial 16S rDNA-ITS genes and the V3-V4 16SrDNA gene, respectively. A Maximum Likelihood tree analysis was performed using the edge-linked partition model in IQ-TREE v.1.6.12^49^ with 1000 replicates of the fast standard nonparametric bootstrap. Bayesian inference analysis was carried out using the Markov chain Monte Carlo process for 20,000,000 generations, retaining one tree in every 1,000 generations, and the first 10% of each tree was discarded using the MrBayes 3.2.7a program. Trees were visualized in Figtree v1.4.2.

### Measurement of cyanobionts cell size

The cell size of 30 individual cyanobionts were measured. One to two cyanobionts per host cell were randomly selected on the micrographs of the *Ornithocercus* host with cyanobionts. Sizes were measured using the software AxioVision SE64 Rel. 4.8.

## Supplementary information

Supplementary Information.

## Data Availability

The partial 16S rDNA- ITS sequences in four types of *Ornithocercus* cyanobionts were deposited in GenBank under the accession numbers MW557373-MW557376. The raw sequence data obtained through the MiSeq platform were deposited in the NCBI Sequence Read Archive (SRA) database with the BioSample accession ID SAMN17602147-SAMN17602284 under the BioProject PRJNA695134. The alignment data of both OTU sequences and the cyanobacterial partial 16S rDNA-entire TIS gene, and data of relative abundance of *Ornithocercus* symbionts were listed in 10.6084/m9.figshare.14132522.v1.
