# Supplementary material for: Cyanobiont genetic diversity and host specificity of cyanobiont-bearing dinoflagellate Ornithocercus in temperate coastal waters
Source: Sci Rep. 2021 May 4;11:9458. doi: 10.1038/s41598-021-89072-z (PMC8097063; doi:10.1038/s41598-021-89072-z)
Supplement: Supplementary file 1 — Supplementary Information. [file 41598_2021_89072_MOESM1_ESM.docx]

**SUPPLEMENTARY INFORMATION**

**Cyanobiont genetic diversity and host specificity of cyanobiont-bearing dinoflagellate *Ornithocercus* in temperate coastal waters**

**Miran Kim^1,2^, Dong Han Choi^3^, Myung Gil Park^4,*^**

^1^Research Institute for Basic Sciences, Chonnam National University, Gwangju 61186, Republic of Korea,

^2^ Honam National Institute of Biological Resources, 99 Gohadoangil, Mokpo 587262, Republic of Korea^3^Marine Ecosystem Research Center, Korea Institute of Ocean Science and Technology, 385 Haeyangro, Yeongdogu, Busan 49111, Republic of Korea

^4^Department of Oceanography, Chonnam National University, Gwangju 61186, Republic of Korea7

| Target | Primer | Oligonucleotide sequence (5'-3') | Remarks | Reference |
| --- | --- | --- | --- | --- |
| V3-V4 regions of 16S rDNA | Pro341F | TCGTCGGCAGCGTCAGATGTGTATAAGA  GACAG**CCTACGGGNSGCWGCAG** | Forward primer for 1st-round PCR for MiSeq | Modified Takahashi et al. 2014 |
|  | Pro805R | GTCTCGTGGGCTCGGAGATGTGTATAAG  AGACAG**GACTACNVGGGTATCTAAT** | Reverse primer for 1st-round PCR for MiSeq |  |
|  | CYA359F | TCGTCGGCAGCGTCAGATGTGTATAAGA  GACAG**GGGGAATYTTCCGCAATGGG** | Forward primer for 1st-round PCR for MiSeq | Nübel et al. 1997 |
|  | CAY781R | GTCTCGTGGGCTCGGAGATGTGTATAAG  AGACAG**GACTACAGGGGTATCTAATCCCTT** | Reverse primer for 1st-round PCR for MiSeq |  |
| Illumina index | i5 | AATGATACGGCGACCACCGAGATCTACAC | Forward primer for nested PCR |  |
|  | i7 | CAAGCAGAAGACGGCATACGAGAT | Reverse primer for nested PCR |  |
| partial 16S rDNA-entire ITS genes | U16F1 | AGAGTTTGATCCTGGCTCAG | Forward primer for 1st PCR | Yoon et al. 2009 |
|  | ITS-br | CCGTGAGCCCTTTGTAGCTTG | Reverse primer for PCR/  Sequencing reverse primer | Lavin et al. 2008 |
|  | CYA106F | CGGACGGGTGAGTAACGCGTGA | Forward primer for 2nd PCR | Nübel et al. 1997 |
|  | CYA359F | GGGGAATYTTCCGCAATGGG | Sequencing forward primer | Nübel et al. 1997 |

**Supplementary Table S1.** Primers used in this study. Underlined regions indicate Illumina over hang adapter sequences. Bold text indicates PCR primer sequences.

**Supplementary Table S2.** GenBank accession numbers used in the phylogenetic tree of Fig. 3 and 7.

| Taxon | 16SrDNA | ITS sequence |
| --- | --- | --- |
| *Synechococcus* sp. WH5701 | AY172832 | AF397729 |
| *Synechococcus* sp. RS9907 | AY172817 | JF306818 |
| *Synechococcus* sp. WH8012 | AF539812 | AF397709 |
| *Synechococcus* sp. WH8002 | AY172833 | AF397707 |
| *Synechococcus* sp. WH8109 | AY172836 | AF397710 |
| *Synechococcus* sp. RS9905 | AY172815 | JF306816 |
| *Synechococcus* sp. WH8102 | BX569694 | AF397712 |
| *Synechococcus* sp. WH8103 | LN847356 | AF397713 |
| *Synechococcus* sp. WH8016 | AY172834 | AF397718 |
| *Synechococcus* sp. WH8020 | AY172835 | AF397719 |
| *Synechococcus* sp. UW105 | JQ421034 | DQ351310 |
| *Synechococcus* sp. KORDI-100 | CP006269 | KC192550 |
| *Synechococcus* sp. WH7805 | AF001478 | AF397721 |
| *Synechococcus* sp. KORDI-53 | FJ497736 | FJ497764 |
| *Synechococcus* sp. RCC1020 | JF306719 | JF306776 |
| *Synechococcus* sp. RCC66 | JF306679 | JF306808 |
| *Synechococcus* sp. UW179 | JQ421033 | JQ421041 |
| *Synechococcus* sp. UW180 | JQ421032 | JQ421042 |
| Cyanobiont of *Amphisolenia bidentata* clone APac | AY444914 |  |
| Cyanobiont of *Amphisolenia bidentata* clone BAtl | AY444915 |  |
| Cyanobiont of *Amphisolenia globifera* clone BAtl-f | AY444918 |  |
| Cyanobiont of *Amphisolenia* *globifera* clone D-1Atl | AY444919 |  |
| Cyanobiont of *Amphisolenia* *globifera* clone D-2Atl | AY444920 |  |
| Cyanobiont of *Amphisolenia* *globifera* clone E-1Atl | AY444921 |  |
| Cyanobiont of *Amphisolenia* *globifera* clone E-2Atl | AY444922 |  |
| Cyanobiont of *Amphisolenia globifera* clone E-3Atl | AY444923 |  |
| Cyanobiont of *Amphisolenia* *bidentata* clone FAtl-f | AY918885 |  |
| Cyanobiont of *Citharistes* sp. clone BAtl-f | AY444925 |  |
| Cyanobiont of *Citharistes* sp. clone CAtl-f | AY444926 |  |
| Cyanobiont of *Citharistes* sp. clone D-1Atl | AY444927 |  |
| Cyanobiont of *Citharistes* sp. clone D-2Atl | AY444928 |  |
| Cyanobiont of *Citharistes* sp. clone E-1Atl | AY444929 |  |
| Cyanobiont of *Citharistes* sp. clone E-2Atl | AY444930 |  |
| Cyanobiont of *Citharistes* sp. clone E-3Atl | AY444931 |  |
| Cyanobiont of *Citharistes* sp. clone FAtl-f | AY444932 |  |
| Cyanobiont of *Ornithocercus* sp. clone AAtl | AY444956 |  |
| Cyanobiont of *Ornithocercus* sp. clone BAtl | AY444957 |  |
| Cyanobiont of *Ornithocercus* sp. clone CPac | AY444960 |  |
| Cyanobiont of *Ornithocercus* sp. clone DPac | AY444961 |  |
| Cyanobiont of *Ornithocercus* sp. clone HAtl | AY444965 |  |
| Cyanobiont of *Ornithocercus* sp. clone EPac | AY444962 |  |
| Cyanobiont of *Ornithocercus* sp. clone FPac | AY444963 |  |
| Cyanobiont of *Ornithocercus* sp. clone GAtl | AY444964 |  |
| Cyanobiont of *Ornithocercus* sp. clone IAtl | AY444966 |  |
| Cyanobiont of *Ornithocercus magnificus* OmCyn01 | BIMP01000001 | BIMP01000001 |
| Cyanobiont of *Histioneis* sp. clone A-2Atl | AY444939 |  |
| Cyanobiont of *Histioneis* sp. clone A-3Atl | AY444940 |  |
| Cyanobiont of *Histioneis* sp. clone B-1Atl | AY444941 |  |
| Cyanobiont of *Histioneis* sp. clone CAtl | AY444943 |  |
| Cyanobiont of *Histioneis* sp. clone Dpac | AY444944 |  |
| Cyanobiont of *Histioneis* sp. clone Epac | AY444945 |  |
| Cyanobiont of *Histioneis* sp. clone Fpac | AY444946 |  |
| Cyanobiont of *Histioneis* sp. clone Hpac | AY444949 |  |
| Cyanobiont of *Histioneis* sp. clone IAtl | AY444950 |  |
| Cyanobiont of *Histioneis* sp. clone Jpac | AY444951 |  |
| Cyanobiont of *Histioneis* sp. clone Kpac | AY444952 |  |
| Cyanobiont of *Histioneis* sp. clone L-1Pac | AY444953 |  |
| Cyanobiont of *Histioneis* sp. clone L-2Pac | AY444954 |  |
| Cyanobiont of *Histioneis* sp. clone L-3Pac | AY444955 |  |
| *Prochlorococcus* sp. MIT9313 | AF053399 | AF397704 |
| *Prochlorococcus* sp. MIT9303 | AF053397 | AF397703 |
| *Prochlorococcus marinus* SB | AF001473 | AF397693 |
| *Prochlorococcus marinus* MIT9202 | AF115269 | AF397683 |
| *Prochlorococcus marinus* AS 9601 | CP000551 | AF397677 |
| *Prochlorococcus marinus* NATL2A | AF311219 | AF397695 |
| *Prochlorococcus marinus* NATL1A | CP000553 | AF397694 |
| *Prochlorococcus marinus* SS2 | NZ_JNAY01000018 | AF397698 |
| *Prochlorococcus marinus* MIT9211 | AF115270 | AF397702 |

| Temperature (°C) | | Station | | | | | | | | | |
| --- | --- | --- | --- | --- | --- | --- | --- | --- | --- | --- | --- |
| Date | Depth (m) | W1 | W2 | W3 | W4 | W5 | W6 | W7 | W8 | W9 | Pohang |
| Mar 2017-2019 | 0 | 11.1± 0.1 | 11.3± 0.7 | 12.9± 0.7 | 13.2± 0.4 | 13.7± 0.2 | 14.2± 0.3 | 14.4± 0.2 | 14.7± 0.3 | 14.4± 0.2 |  |
|  | 30 | 11.0± 0.1 | 11.2± 0.4 | 12.9± 0.7 | 13.1± 0.8 | 13.5± 0.4 | 14.0± 0.4 | 14.1± 0.4 | 14.3± 0.1 | 14.3± 0.2 |  |
| Jun 2017-2019 | 0 | 18.8± 1.8 | 17.5± 1.1 | 18.8± 2.3 | 19.5± 1.4 | 21.8± 1.1 | 21.5± 0.9 | 21.8± 0.8 | 21.2± 1.6 | 19.7± 1.5 |  |
|  | 30 | 16.5±2.2 | 14.8± 1.6 | 14.4± 1.3 | 14.7± 1.2 | 15.2± 0.7 | 17.0± 1.3 | 17.5± 1.1 | 18.0± 0.4 | 16.9± 1.1 |  |
| Sep 2017-2019 | 0 | 22.4± 0.9 | 22.2± 1.5 | 23.0± 0.9 | 23.5± 1.9 | 24.4± 1.3 | 24.5± 2.1 | 25.2± 1.8 | 25.2± 1.8 | 24.5± 2.4 |  |
|  | 30 | 21.9± 1.1 | 21.1± 1.6 | 20.0± 2.5 | 20.1± 2.5 | 20.0± 3.2 | 20.3± 3.8 | 21.1± 3.5 | 23.1± 1.0 | 23.4± 2.4 |  |
| Nov 2017-2019 | 0 | 14.5± 0.3 | 14.8± 0.5 | 15.2± 1.0 | 15.7± 1.3 | 16.6± 1.3 | 18.4± 0.4 | 18.7± 0.3 | 19.1± 0.9 | 18.9± 0.7 |  |
|  | 30 | 14.4± 0.2 | 14.6± 0.5 | 15.2± 1.0 | 15.5± 1.2 | 15.9± 1.1 | 18.7± 0.5 | 18.9± 0.5 | 19.3± 0.8 | 18.8± 0.6 |  |
| Dec 2017 | 0 |  |  |  |  |  |  |  |  |  | 11.7 |
|  |  |  |  |  |  |  |  |  |  |  |  |
| Salinity |  | Station | | | | | | | | | |
| Date | Depth (m) | W1 | W2 | W3 | W4 | W5 | W6 | W7 | W8 | W9 | Pohang |
| Mar 2017-2019 | 0 | 33.9± 0.2 | 33.8± 0.3 | 34.3± 0.0 | 34.2± 0.1 | 34.4± 0.2 | 34.0± 0.6 | 34.5± 0.2 | 34.4± 0.1 | 34.4± 0.1 |  |
|  | 30 | 33.9± 0.2 | 34.0± 0.2 | 34.3± 0.4 | 34.3± 0.2 | 34.4± 0.2 | 34.4± 0.1 | 34.4± 0.2 | 34.5± 0.2 | 34.4± 0.1 |  |
| Jun 2017-2019 | 0 | 33.6± 0.1 | 33.6± 0.2 | 33.5± 0.3 | 33.0± 0.2 | 32.5± 0.1 | 32.5± 0.3 | 33.0± 0.4 | 33.1± 0.5 | 33.5± 0.4 |  |
|  | 30 | 33.6± 0.2 | 33.6± 0.2 | 33.7± 0.2 | 33.6± 0.2 | 33.8± 0.4 | 33.7± 0.3 | 33.7± 0.1 | 33.7± 0.2 | 33.9± 0.0 |  |
| Sep 2017-2019 | 0 | 32.3± 0.4 | 32.2± 0.5 | 32.0± 0.8 | 31.7± 1.2 | 31.3± 1.9 | 31.8± 1.3 | 32.0± 1.3 | 31.9± 1.4 | 31.8± 1.5 |  |
|  | 30 | 32.4± 0.4 | 32.5± 0.3 | 32.6± 0.3 | 32.4± 0.8 | 32.7± 1.1 | 32.9± 1.2 | 32.6± 1.0 | 32.5± 1.2 | 32.4± 0.1 |  |
| Nov 2017-2019 | 0 | 32.5± 0.1 | 33.6± 0.2 | 33.4± 0.2 | 33.4± 0.4 | 33.5± 0.5 | 33.8± 1.4 | 34.0± 0.1 | 34.0± 0.3 | 34.1± 0.1 |  |
|  | 30 | 33.2±0.1 | 33.2± 0.2 | 33.4± 0.2 | 33.54± 0.4 | 33.7± 0.5 | 34.0± 0.1 | 34.0± 0.1 | 34.0± 0.1 | 34.1± 0.1 |  |
| Dec 2017 | 0 |  |  |  |  |  |  |  |  |  | 34.3 |

**Supplementary Table S3.** Temperatures and salinities at the surface and depth of 30 m in the study areas. Data were presented as mean with standard deviation (SD) of the mean.

| *Ornithocercus* cyanobionts | partial 16S rDNA | | | entire ITS | | |
| --- | --- | --- | --- | --- | --- | --- |
|  | Size (bp) | Type 2 | Type 3 | Size (bp) | Type 2 | Type 3 |
| Type 2 | 1140 | - | - | 697 | - | - |
| Type 3 | 1140 | 2.63 | - | 708 | 3.73 | - |

**Supplementary Table S4.** Genetic p-distance matrix of a partial 16S rDNA and entire ITS gene among two genetic types 2 and 3 of Ornithocercus cyanobionts obtained


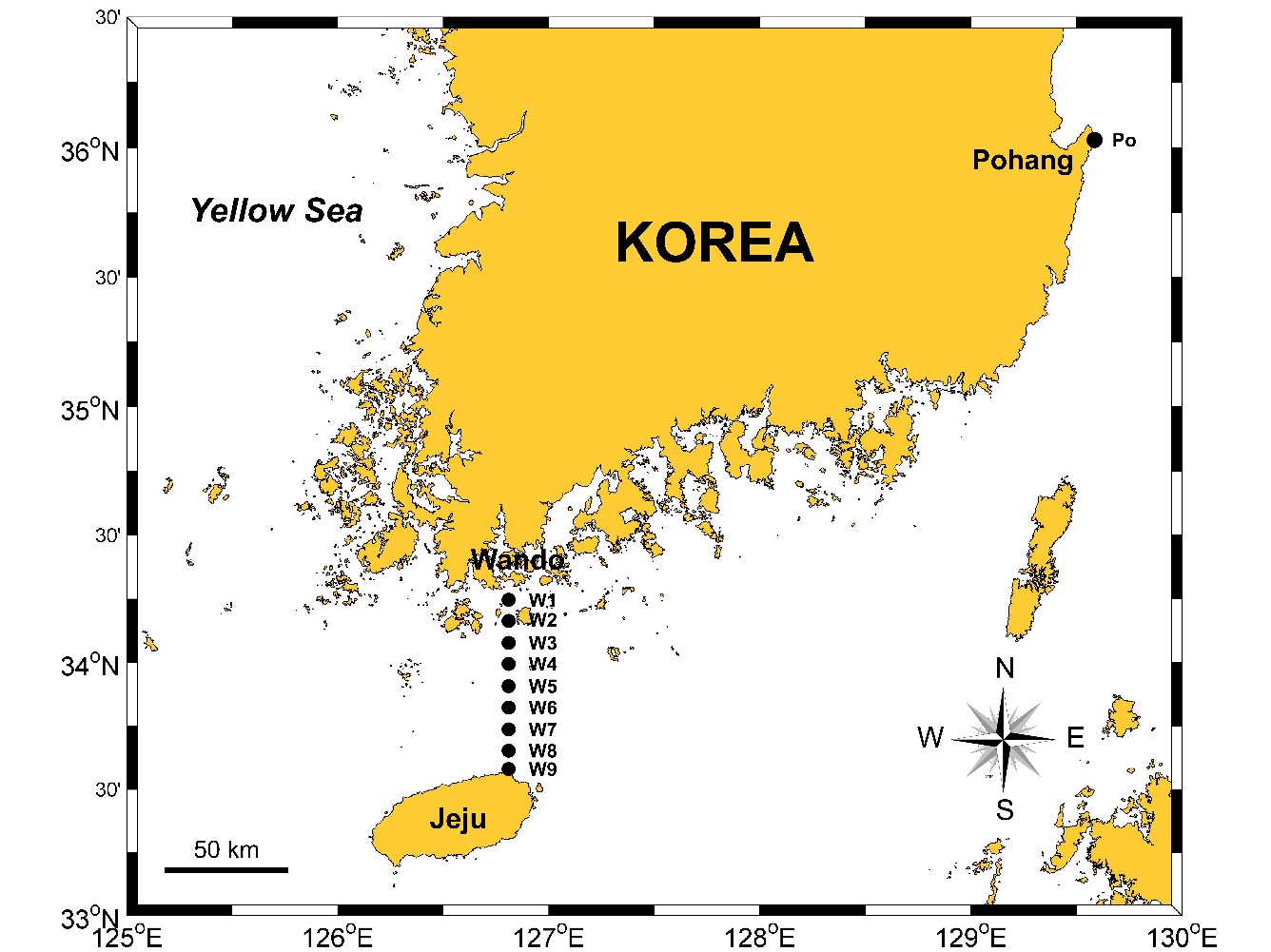


**Supplementary Figure S1.** Location of sampling stations. The South Sea of Korea transects between Wando and Jeju (Stans W1 to W9) and coastal area of Pohang (Po). The map was generated using the Matlab (Version R2009) software package.


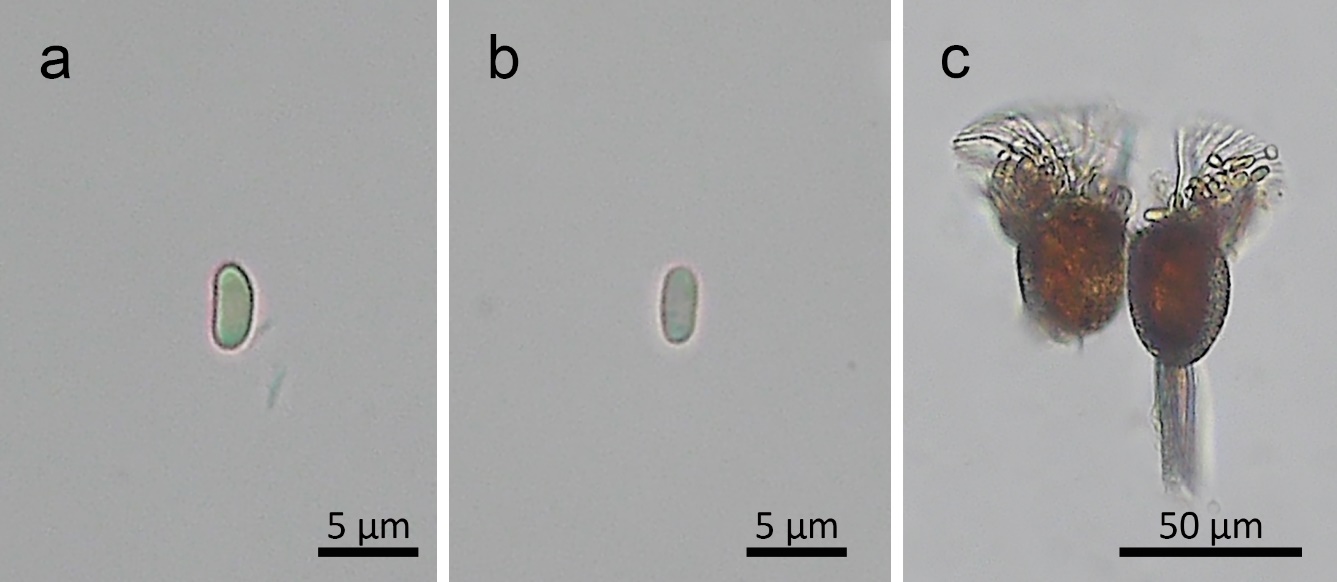


**Supplementary Figure S2.** Micrographs of the cyanobionts of Type 1b (a) and Type 2 (b) and dividing *Ornithocercus* host cell (c). The cyanobionts used for the single-cell PCR were isolated from the live *Ornithocercus* hosts, respectively. The dividing host cell was observed in the fixed field sample.
